# The post-diapause vibrational behavior, motility, and survival of the brown marmorated stink bug *Halyomorpha halys* (Stål) adults at different temperatures

**DOI:** 10.1038/s41598-023-50480-y

**Published:** 2024-01-12

**Authors:** Jalal M. Fouani, Marica Scala, Valentina Zaffaroni-Caorsi, Vincenzo Verrastro, Gianfranco Anfora, Valerio Mazzoni

**Affiliations:** 1 Center Agriculture Food Environment, University of Trento, 38010 San Michele All’Adige, Italy; 2 Department of Environmental and Earth Sciences, University of Milano Bicocca, 20126 Milano, Italy; 3 CIHEAM Bari - International Centre for Advanced Mediterranean Agronomic Studies, Via Ceglie 9, 70010 Valenzano, Italy; 4 Research and Innovation Centre, Fondazione Edmund Mach, 38010 San Michele All’Adige, Italy

**Keywords:** Ecology, Behavioural ecology, Climate-change ecology, Invasive species

## Abstract

Substrate-borne vibrational communication is common in pentatomids. Although several works exist on the vibrational communication of *Halyomorpha halys*, its vibrational behavior post diapause has not been investigated. In this study, we recorded *H. halys* overwintered adults using laser doppler vibrometers at three temperatures: 10 °C (inactivity), 18 °C (breaking of diapause), and 25 °C (peak of mating activity). The aim was to assess the effect of temperature on the signaling, motility, and survival of *H. halys*. The insects were sexed into different cages and recorded separately or joined with a cage of the opposite sex. We calculated the total time spent on signaling and walking per replica. The males predominantly emitted male signal 1 (MS1) throughout the four months of recordings. The females exclusively emitted female signal 2 (FS2) when joined with the opposite sex cage the first two months of recordings. Interestingly, they also started FS2 signaling when recorded separately, after two months. No signaling was recorded at 10 °C. At 25 °C, the signaling latency time before vibrational signaling was 24 h compared to 23 days at 18 °C. The short latency time at 25 °C correlated with a higher death rate in early stages of recording. Male walking activity was significantly higher in joined cages at 18 °C and 25 °C, suggesting the increased searching behavior near the opposite sex. Overwintered *H. halys* could adapt to different conditions whereas low temperatures maintain the diapause which is characterized by no signaling activity. Our results provide a foundation for bioclimatic modeling of climate change effects on *H. halys* and insights into the use of vibrational playbacks for mass trapping and monitoring as control techniques.

## Introduction

In recent years, the introduction of alien insect pests into new areas has caused major economic damage to agriculture. These intrusions are generally due to anthropic activities such as global trading and increased human travel^1,2^. On a worldwide scale, biological invasions are responsible for weighty economic losses estimated at approximately US$ 1.288 trillion (2017 US dollars), in addition to significant biodiversity declines^3^. *Halyomorpha halys* (Heteroptera: Pentatomidae) is among these intruders. This Asian species is a notorious invasive pest that has emerged as a phytosanitary threat, causing significant economic losses to crops in Europe and the United States of America (USA)^4^. The spread of *H. halys* first started with its accidental introduction into North America and Europe, and at present, its expansion has reached at least 41 different countries^5^. The invasiveness of this pest is ensured by its high dispersal capabilities^6^ and hitchhiker behavior, as it can exploit cargo shipments or other transportation means for passive movement^7^. Moreover, given their strong flying capacity, the adults display an escape behavior when disturbed, making the effectiveness of chemical control with pesticide applications uncertain^8^. Recently, more efforts have been made with respect to sustainable control of *H. halys*, mainly exploiting the potential effectiveness of native and/or exotic parasitoids and their potential inclusion within classical biological control programs^9–11^. Furthermore, higher attention is being dedicated to research on other important aspects of *H. halys* biology, including vibrational communication^12,13^. Biotremology is a recently recognized scientific discipline^14^ that has shown great potential for its application in control programs to manage various pests and vectors^15–19^. Interestingly, applied biotremology techniques proved to be efficient as control measures against *H. halys*^20^. In the Pentatomidae family, vibrational communication is common and mediates short-range communication during mating behavior^21^. In a typical scheme, females begin emitting low-frequency vibrational signals once prompted by male pheromones^22^. Males respond with their own signals^21^ while producing larger pheromone quantities^23^, and will then start searching for a stationary female that is emitting her signal^24,25^. Regarding *H. halys*, males and females emit sex-specific courtship signals^12^. Male Signal 1 (MS1) is a relatively long vibrational emission with a peak frequency in the range of 50 Hz, whereas Male Signal 2 (MS2) consists of a train of short pulses with a decreasing frequency modulation from 85 to 40 Hz^12^. Female signals have similar characteristics and are thus called Female Signals 1 (FS1) and 2 (FS2). The dynamics of signal emission are largely unknown, however, the main known difference between sexes lies in males emitting their signals spontaneously, even if alone, whereas females emit their signals only in the presence of other individuals (e.g., responding to male signals). Furthermore, males predominantly emit MS1, while females mostly emit FS2^12^. Under natural conditions, the mating behavior of *H. halys* occurs from spring, after overwintering when the species survives in a diapause state, to late summer^8,26^. Temperature is a key parameter that regulates the activity of *H. halys*^27^. In particular, under 14 °C the adults tend to stay within the wintering phase while the peak egg-laying activity takes place between 23.5 and 28.4 °C^26^. According to Fisher et al.^28^, events including high temperatures and low humidity can significantly reduce *H. halys* survival over time. Most studies evaluating the effects of temperature on this species have focused on the reproductive season; hence, little is known on how these insects recommence their vibrational signaling in the post-dormancy phase and how it can affect their survival.

In the present study, we examined the influence of three different temperatures (10 °C, 18 °C, and 25 °C) on restoring the vibrational calling activity and motility of *H. halys* adults for a better understanding of their phenology after diapause. Given that 14 °C is the threshold above which the diapause is broken, we chose 10 °C as the negative control in the range of temperatures corresponding to inactivity. In contrast, 18 °C and 25 °C were chosen in temperature ranges of suboptimal (i.e., 14–23.5 °C) and optimal mating and egg-laying activity (i.e., 23.5–28.4 °C). Our research aimed to describe the post-diapause vibrational behavior and motility of overwintered *H. halys* adults at these temperatures. Additionally, we examined the survival of the insects and correlated it with the recorded vibrational communication at each temperature. Given the gap of knowledge on the sex-related vibrational emission of *H. halys* adults, the insects were recorded in their respective cages within different settings for each temperature treatment. As a result, the recordings were performed on control cages (i.e., five males and five females together, hereinafter “CCg”), single cages (hereinafter “SCg”), and joined cages (hereinafter “JCg”). The latter recording setting was achieved by clipping single cages of opposite sexes on the side.

## Results

The experimental differences resulting from the different cage state recording settings are summarized in Table 1.

**Table 1:** Summary of experimental differences resulting from cage recording settings.

Cage state treatment	Copulation	Vibrational signals	Pheromones
Control (CCg)	Can occur	Perceived	Perceived
Single (SCg)	Cannot occur	Not perceived	Not perceived
Joined (JCg)	Cannot occur	Perceived	Perceived

**Figure 1:**
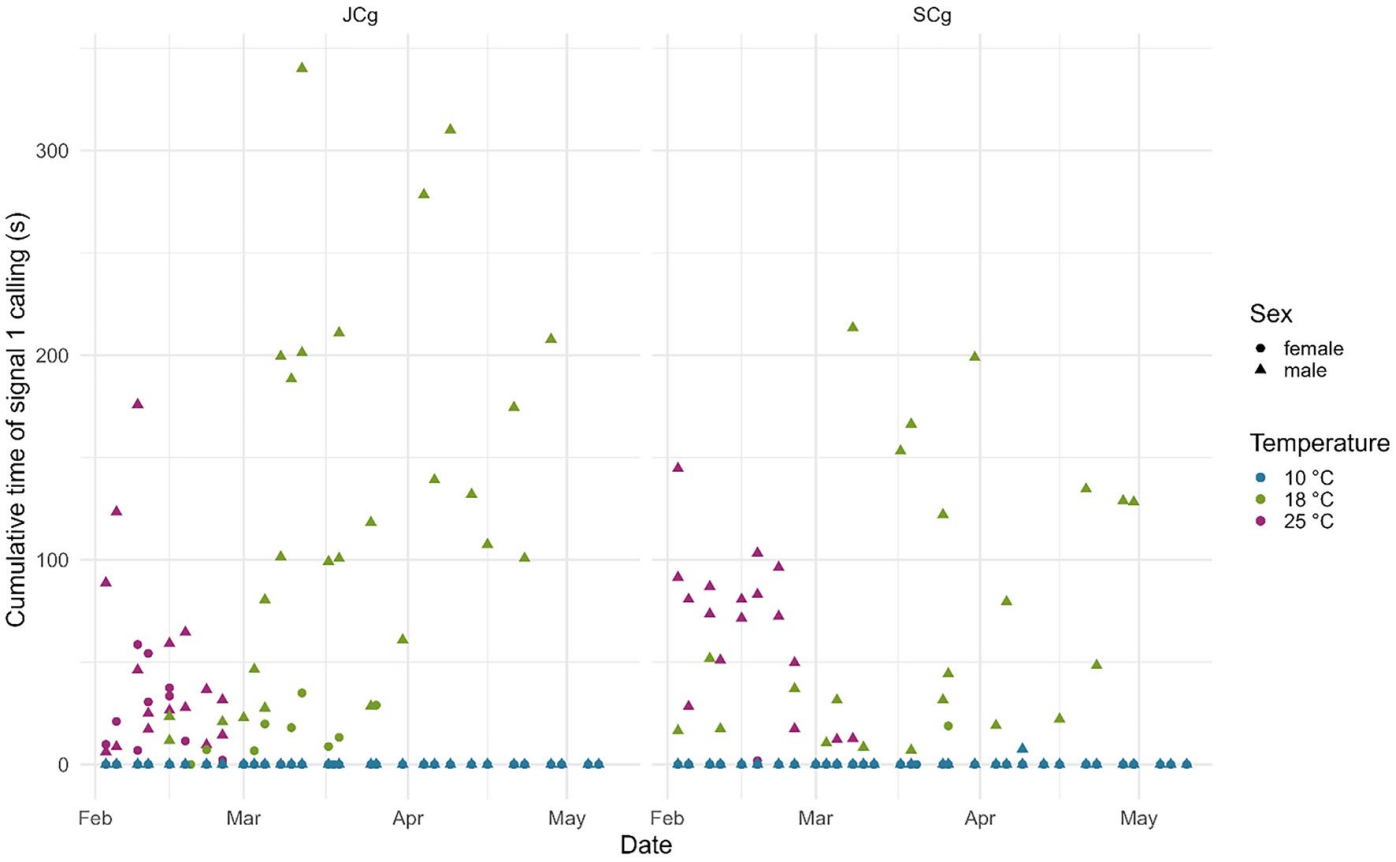
Cumulative Signal 1 emission for each recording of male and female *Halyomorpha halys* in joined (JCg) and separated (SCg) cages at all temperatures.

**Figure 2:**
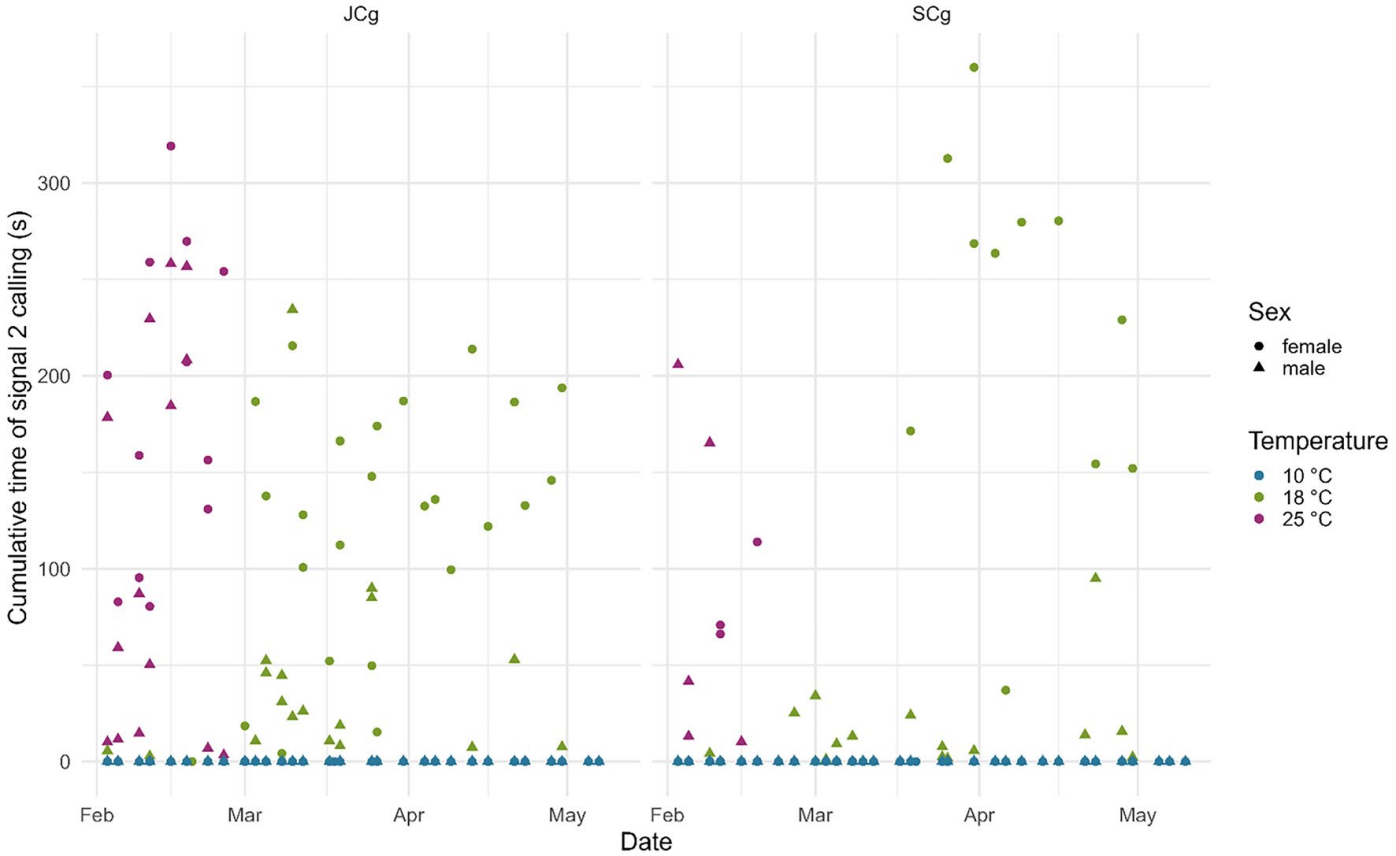
Cumulative Signal 2 emission for each recording of male and female *Halyomorpha halys* in joined (JCg) and separated (SCg) cages at all temperatures.

**Figure 3:**
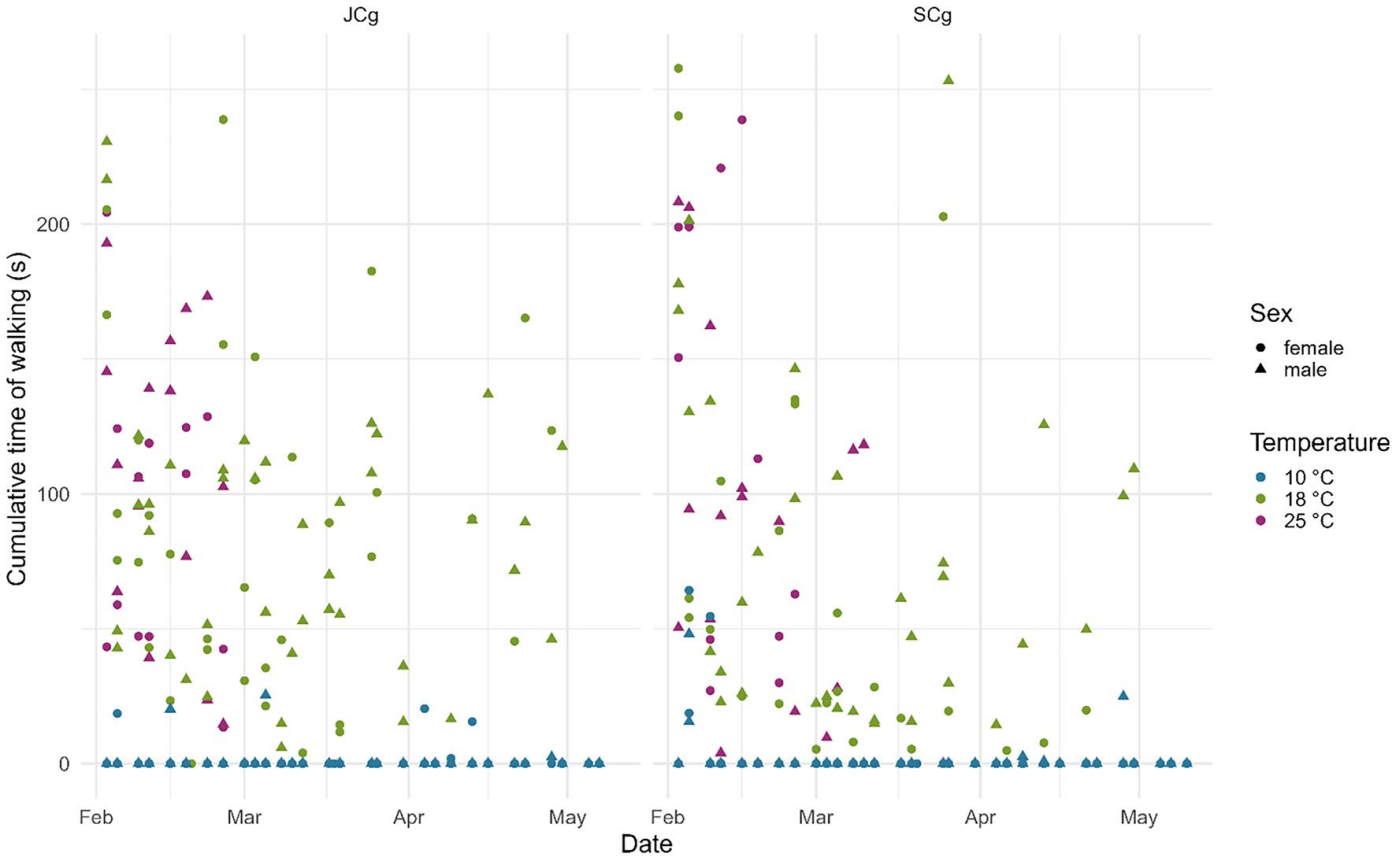
Cumulative walking time for each recording of male and female *Halyomorpha halys* in joined (JCg) and separated (SCg) cages at all temperatures.

The temperature had various effects on the cumulative periods of calling and motility (measured as vibrational noise associated with walking). At 10 °C, no signaling activity of any type was registered, while a minimum amount of motility was observed (Figs. 1, 2, 3). In contrast, 18 °C and 25 °C were the temperatures at which *H. halys* was active; however, some discrepancies were found in signal calling between the two temperatures. Within the 18 °C treatment, females emitted FS2 prevalently when they were recorded in JCg. However, they were also found to be active when recorded in SCg, especially from mid-March onwards, accounting for a delay of 15 days compared with JCg (Fig. 2). Moreover, they only started signaling 35 days after diapause, while the males were found to be signaling within the first week after diapause. Within the 25 °C treatment, both males and females were active immediately after diapause (01 days). The effect of temperature on signaling was also statistically evident as the significant factors of signal 1’s GLMM were temperature 18 °C (z = 0.923, *P *= 0.00656), temperature 25 °C (z = 2.14277, *P *= 0.02641), and sex male (z = 4.928, *P *= 8.31e−07) (Table 2). The model further confirmed the higher tendency of males to emit signal 1 compared to females (Fig. 1 & Fig. S2). The significant factors of signal 2’s GLMM were temperature 18 °C (z = 15.935, *P *= 2e−16), temperature 25 °C (z = 19.653, *P* = 2e−16), and sex male (z = − 4.049, *P* = 5.13e−05) (Table 2). The model confirmed the higher tendency of females to emit signal 2 compared to males (Fig. 2 & Fig. S2). The significant factors of motility’s GLMM were temperature 18 °C (z = 4.380, *P* = 1.19e−05) and temperature 25 °C (z =5.004, *P* = 5.62e−07) (Table 2).

**Table 2:** Results of generalized linear mixed-effects model (GLMM) for signaling and walking.

A	Model synthesis	AIC	BIC	LogLik	Dev	df	Distribution
(Signal 1)	Signal1 ~ temperature + sex + state + (1|cage)	1358.3	1412.8	− 666.1	1332.3	477	Negative binomial
(Signal 2)	Signal2 ~ temperature + sex + state + (1|cage)	1497.1	1547.5	− 736.6	1437.1	477	Tweedie
(Motility)	walking ~ temperature + state + (1|cage)	2600.9	2651.2	− 1288.5	2576.9	478	Tweedie

B	Fixed effects	Estimate	Std. Error	Z value	Pr ( >|z|)		
(Signal 1)	(Intercept)	0.90958	0.98523	0.923	0.35589		
	Temperature 18 °C	2.61910	0.96350	2.718	0.00656 **		
	Temperature 24 °C	2.14277	0.96513	2.220	0.02641 *		
	Sex male	1.09746	0.22270	4.928	8.31e−07 ***		
(Signal 2)	(Intercept)	− 3.695e+01	6.116e+03	− 0.006	0.995		
	Temperature 18 °C	4.586	2.878e−01	15.935	< 2e−16 ***		
	Temperature 24 °C	5.516	2.807e−01	19.653	< 2e−16 ***		
	Sex male	− 1.251	3.090e−01	–4.049	5.13e−05 ***		
(Motility)	(Intercept)	2.86618	0.36129	7.933	2.13e−15***		
	Temperature 18 °C	1.50367	0.34329	4.380	1.19e−05 ***		
	Temperature 24 °C	1.78674	0.35707	5.004	5.62e−07 ***		

A) The best explanatory model for each dependent variable. B) The significant independent variables of the chosen models. Dev (deviance), Est (estimate), df (df residuals). *p < 0.05, **p < 0.01, ***p < 0.001.

**Figure 4:**
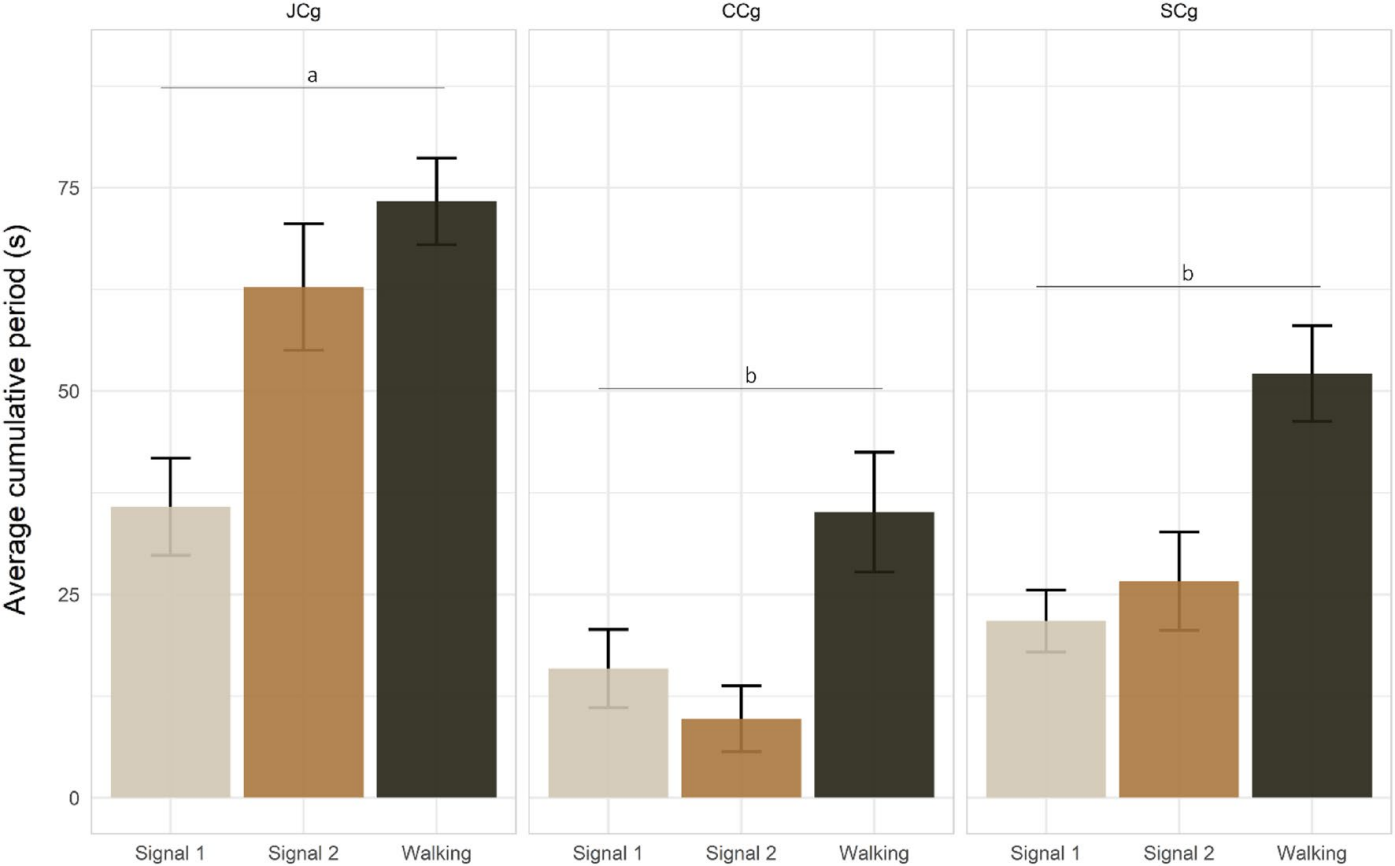
Barplots showing the mean signaling and walking differences when cages were recorded separately (SCg) or joined (JCg) vs the control (CCg). Recording condition groups indicated by different letters show significant differences (Wilks’ Lambda type non-parametric interference at *p* < 0.001). The error bars represent the standard errors.

Regarding cage state treatment, our results indicated a significant effect of copulation inhibition (JCg) on the signaling and motility of *H. halys* (Wilks’ Lambda = 10.010, df = 6, 622, *P *< 0.001) (Fig. 4). In particular, higher values of both signals 1 and 2 emission were recorded for JCg compared to CCg and SCg (Fig. 4). The post-hoc test showed that the cumulative signal emission and motility times were significantly higher for JCg than for CCg and SCg (Fig. 4). Moreover, the females spent significantly higher cumulative periods emitting signal 1 in JCg compared to SCg (χ2 = 12.21, df = 1, *p *< 0.001) (Fig. S2). Males spent higher cumulative periods emitting Signal 1 in JCg; however, the difference was not statistically significant. Both females (χ2 = 10.46, df = 1, *p* < 0.001) and males (χ2 = 7.1891, df=1, *p *< 0.01) spent significantly higher cumulative periods emitting Signal 2 in JCg (Fig. S2). The same was applied to motility, as the highest cumulative walking time was recorded when the cages were joined. Only males spent significantly higher cumulative walking periods in JCg (χ2 = 4.23, df = 1, *p* = 0.04) (Fig. S2).

**Figure 5:**
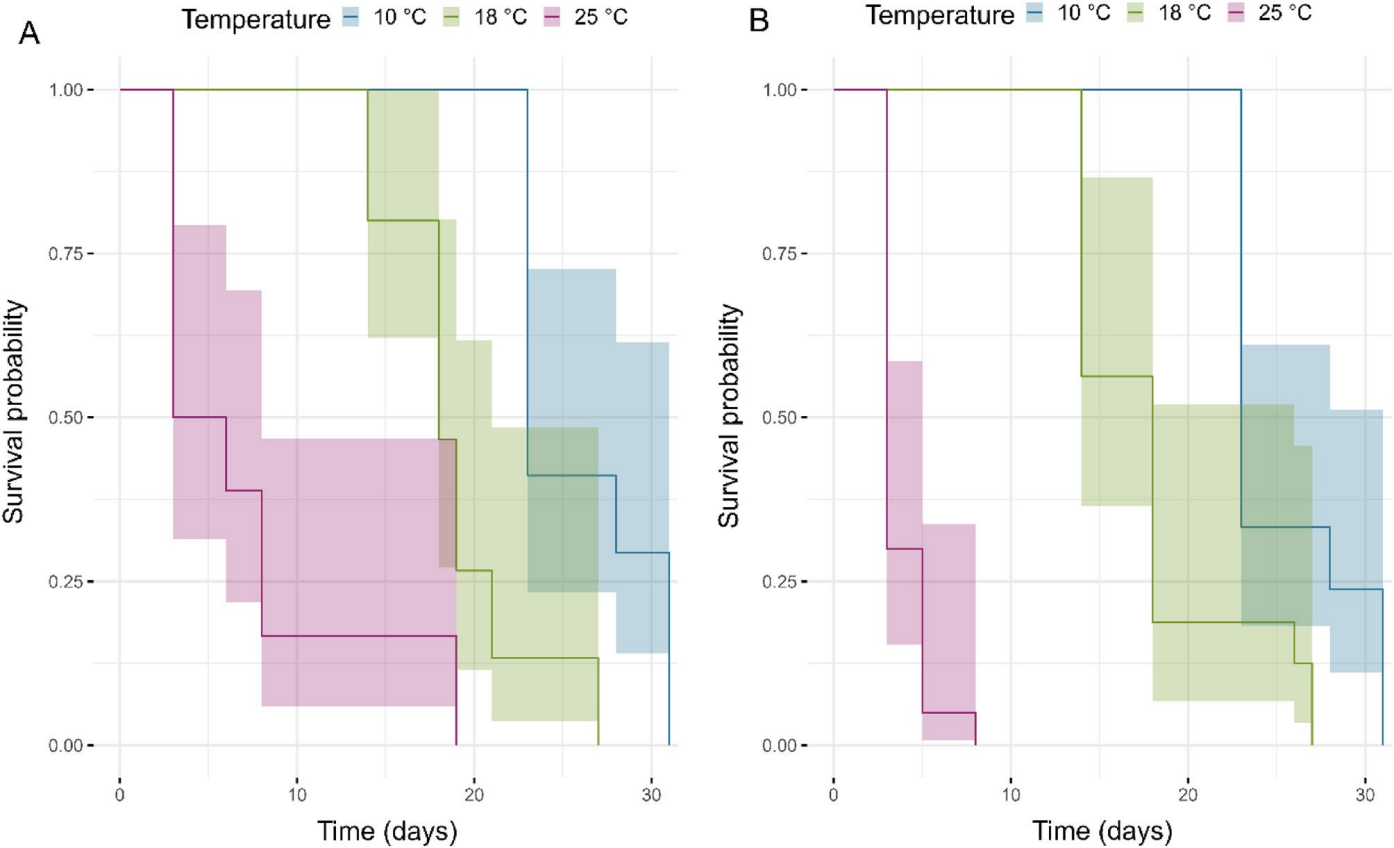
Kaplan–Meier plot describing the survival curves for *Halyomorpha halys* at all temperatures for (**A**) males and (**B**) females. The curves represent the estimated survival probability at each time point. Y-axis shows the probability of being alive at each time point, while X-axis shows the time-points. The shaded areas around the lines represent 95% CI.

To describe insect survival period at each temperature, the Overall Survival (OS) was measured from the date of first observation (day 1) to the date of death (event) until the last follow-up date (day 34). Regarding the overall survival analysis, a total of 107 individuals were considered, with 100% deaths occurring until the end of the study. Looking at the total deaths separated by temperature and sex (Table S1), the median overall survival was higher at 10 °C than at 18 °C and 25 °C. This was further confirmed in the Kaplan-Meier plot, where a lower survival probability was registered at higher temperatures (Fig. 5). Moreover, Table 3 shows the results of the Cox model for males, with a significant difference between the survival at temperature 10 °C against 18 °C (HR = 5.28, 95% CI (2.28–12.2); *p *= 0.0001) and 25 °C (HR = 27.7, 95% CI (12.5–76.6); *p* = 1.54e−10). The survival distribution of events within the female group showed that at 25 °C, all deaths occurred within the first 10 days (Fig. 5B), whereas at 10 °C and 18 °C, the overall survival was higher, with deaths occurring between 3 and 5 weeks.

**Table 3:** Cox regression model for male—temperature; Hazard ratio and 95% confidence interval.

Variables	Hazard ratio (CI 95%)	*p*-value
Ref level 10 °C		
18 °C	5.28 (2.3–12.2)	0.000102***
25 °C	27.7 (10–76.6)	1.54e−10***

*** p < 0.001.

## Discussion

Knowledge of phenology is a key factor in the efficient control of invasive pests. Phenological modeling of *H. halys* suggested that the expansion and population size of this pest are largely prone to temperature and photoperiod^29^. Previous studies have also suggested that diapause is likely initiated and terminated by the photoperiod^30,31^. With this in mind, here the exposure of *H. halys* adults to three different temperatures following their dormancy phase gave further insight into the phenology of this species. In accordance with the literature, we observed a significant effect of temperature on termination of diapause. In addition, given that the photoperiod was fixed for all treatments in our study, temperature might be the abiotic factor of major importance in orchestrating the termination of overwintering. Interestingly, it also appears to play a significant role in restoring the vibrational communication of *H. halys* post-dormancy. Given the previously reported evidence of the photoperiod’s influence on the phenology of *H. halys*, its interaction with temperature and the resulting effect on reinstating vibrational behavior is a theme that deserves further investigation. In our study, the temperatures at which the post-diapause activity and vibrational communication of *H. halys* occurred were consistent with the activity range previously reported in the literature^26^, as the insects only emitted signals at 18 °C and 25 °C. In particular, the MS1 emission was consistent with that previously reported^12^, since males signaled spontaneously even without perceiving female signals (SCg). However, the total signaling time for MS2 was significantly amplified in conditions of joined cages (JCg) at both active temperatures (18 °C and 25 °C). It is imperative to note that within courtship between stink bug adults, multimodal communication could take place including visual stimuli which might in turn result in a higher vibrational communication^32^. As for walking, it is indicative of *H. halys* motility. In fact, higher temperatures (18 °C and 25 °C) were favorable for higher cumulative walking periods, especially for males, which spent significantly longer periods of walking when recorded in JCg. This confirms the mating strategy of males^12^ that dedicate a greater amount of searching behavior when they retrieve nearby female signals to locate them. Our results also confirm that females are significantly more triggered to emit FS2 when MS1 is perceived, that is, when recorded in JCg. The vibrational calling activity differed significantly between the different cage state categories. In fact, within the control cage treatment (CCg), where the insects were not withheld from copulation, we recorded a significantly lower emission of vibrational signals compared with JCg, where insects were physically separated by a net. It should be noted that the main signal type representing this difference was FS2. This could suggest that sexually-mature female *H. halys* in conditions with low likelihood of copulation, might significantly extend the time of their callings to increase their chances of mating.

Regarding the vibrational activity of *H. halys* at 18 °C and 25 °C, we noted some peculiar differences between the two temperatures, which were significantly more apparent in the case of FS2. At 18 °C, FS2 was prevalently emitted in JCg until mid-March, which further confirmed that they were triggered to engage in vibrational communication when they perceived MS1^12^ and pheromones secreted by males^23^. This phenomenon was also observed at 25 °C. However, females emitted FS2 even in the absence of males (SCg) from mid-March onwards at 18 °C, suggesting that they can also emit FS2 spontaneously, albeit with a certain delay with respect to males’ emission of MS1.

Despite this spontaneous calling among females, this phenomenon might have been due to copulation abstinence for over a month after the end of the overwintering phase and/or reaching a certain age. According to Polajnar et al.^12^, females were never found to emit FS2 spontaneously; however, in that study, it was only reported that females were at least seven days old, without specifying whether there were any individuals aged 30 days or older among those tested. In addition, these individuals did not belong to the overwintering generations. In our experiment, the insects were not recorded singularly but in groups of 10 sexed insects per cage, which might have also played a role in eliciting this behavior. For example, the perception of incidental vibrations due to the walking activity could be potentially associated with the presence of conspecifics. The use of incidental vibrations to orientate towards possible prey is well documented in insects and spiders^33^, as well as it is known that aphids react to the approach of a predator by dropping^34^. However, little is known on the interpretation and reaction of phytophagous insects to the incidental vibrations of conspecifics, a theme that deserves further research.

Notwithstanding, it is important to note that we cannot exclude an effect of pheromones because the sexed cages were kept within the same ventilated climatic chamber. In fact, even if pheromones cover an aggregation function in *H. halys* as well as in other stinkbugs, it is also known that their perception can elicit vibrational signaling in males^35,36^. It is also important to note that females at 25 °C, not different from males, were directly active in terms of vibrational calling on the day following the end of diapause. In contrast, it took 35 days for those at 18 °C to become active. This could be attributed to several reasons. First, it suggests that from the end of the diapause, females require time before being vibrationally active, and that this time strongly depends on temperature. Second, females are potentially ready to interact with males for mating immediately after the end of diapause, which means that their reproductive organs do not need any further development. Our results suggest that they are directly active if they encounter favorable conditions immediately after diapause, but their life expectancy will be shorter. Despite active signaling in such conditions, it remains uncertain whether they can reproduce because it is strongly related to the vitellogenic state of females.

Previous studies have suggested that the state of vitellogenesis in female *H. halys* entering overwintering may be related to abiotic factors in the collection area^37^. Moreover, a significant positive correlation was found between collected mated females and the use of pheromone traps^37^. Here, all the studied specimens were collected using pheromone traps, which might indicate that the collected females had already mated prior to entering the overwintering phase. Accordingly, it is important to note that a successful engagement in vibrational duets is only an indicator of mating readiness and does not confirm reproductive maturity, as *H. halys* females may copulate before reaching it^37^.

Given our interest in investigating the post-diapause temperature effect on *H. halys*, the insects were immediately segregated into different temperature treatments after the overwintering phase. This sudden exposure to different temperatures could have affected insect survival rate. Previous studies showed that *H. halys* is susceptible to high temperatures, i.e., up to 36 °C^38^ and even up to 42 °C^28^, whereas the insects fared best at optimal temperatures, such as 25 °C. In this study, the short latency time prior to vibrational calling at 25 °C correlated with a short period of survival, whereas a longer latency time at 18 °C correlated with a longer overall survival of the insects. Because they lived longer, the total signaling activity was higher than that at 25 °C and 10 °C, where no vibrational activity was registered. The individuals we used were overwintered insects, which could explain the discrepancy between our findings and those of Govindan and Hutchison^38^ and Fisher et al.^28^, who used active insects. It is possible that the low overall survival observed at 25 °C is correlated with the abrupt physiological changes that were induced at this temperature. As a result, the insects were immediately ready to start their vibrational communication prematurely. Another explanation could be linked to the energy expenditure for calling emission for mating^39^. In previous studies, signaling effort revealed that indirect costs associated with vibrational signaling had a negative effect on male survival^40,41^.These findings indicate that climate change might affect *H. halys* when encountering high temperatures shortly after diapause. Our results also suggest that the dormancy phase will be shortened with insects becoming prematurely active, but their life expectancy will be shorter.

In view of the above, this study provides further insights into the post-diapause vibrational behavior of *H. halys*. We can conclude that temperature is unquestionably a main factor influencing the emission of vibrational cues, which could lead to significant effects on life span. The sudden exposure at 25 °C resulted in a faster readiness for vibrational calling emission, which led to a shorter lifespan due to energy expenditure for calling emission. Climate change is likely to affect the behavior and distribution of many invasive pests, but further studies of the temperature ranges that influence reproductive behavior are still needed for many species, including *H. halys*. Our results provide further insights into the potential effects of climate change on the vibrational calling activity and motility of *H. halys* after the overwintering phase. Hence, it could set up the basis for further bioclimatic modeling of the climate change effect on *H. halys*, where further studies are needed for extended ranges of suboptimal and extreme temperatures. Moreover, our results grant further insight into biorational control techniques that employ vibrational playbacks for mass trapping and monitoring purposes. That being said, further understanding of the quality of the emitted vibrational signals in different climatic scenarios is required to better replicate them (i.e., playbacks) for monitoring and biorational control techniques. Nevertheless, our results were obtained under laboratory conditions, which may not fully depict what could happen in an open field.

## Methods

### Insect sample and rearing

Adult insects at the verge of dormancy were collected throughout the end of November 2020 within the surrounding hedgerows of Fondazione Edmund Mach, in Northern Italy (46° 11' 34.8" N 11° 08' 08.9" E), using dark live traps as described in Suckling et al.^42^. At the beginning of December 2020, they were transferred to artificial overwintering conditions (i.e., 9 °C; RH 65 ± 5%) for a 7-week period, which is essential for obtaining sexually mature *H. halys* adults^43^. After this 7-week period, the insects were randomly segregated into three climatic chambers (Angelantoni Test Technologies Srl, Italy). Each chamber was set at one of the chosen temperatures (10 °C, 18 °C or 25 °C), with a constant RH of 65 ± 5% and a fixed photoperiod of 16L: 8D. A data logger (model EL-USB-2, Lascar electronics, Whiteparish, United Kingdom) was employed beside the cages to monitor the temperature (ºC) within each climatic chamber. The tested individuals for each temperature treatment consisted of five insect cages, four of which contained at least ten (12 maximum) sexed adults in each cage, corresponding to two male cages and two female cages (Fig. S1). The fifth cage corresponded to the control cage (CCg) containing five males and five females together, hence, without any copulation prevention (Fig. S1). Consequently, the total number of the experimented insects was 168 individuals. The insects were maintained in 22 × 22 × 22 cm fine mesh cages (BugDorm^®^, MegaView, Taiwan) on green beans (*Phaseolus vulgaris* L.), cashew nuts (*Anacardium occidentale*), and carrots (*Daucus carota* subsp. *sativus*). Food was replaced once a week and water was added ad libitum as soaked cotton.

### Insect recording

**Figure 6:**
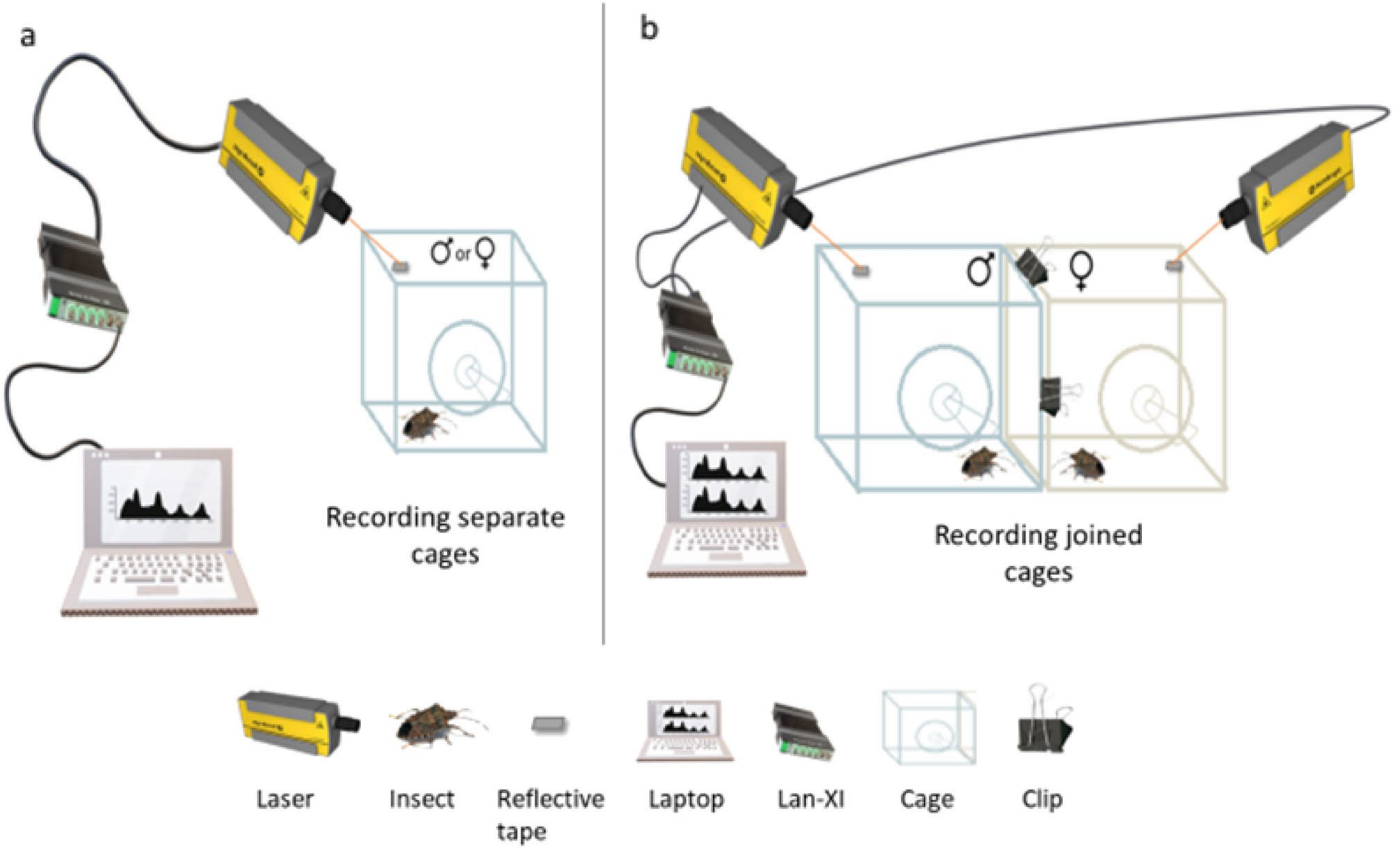
Scheme of the experimental setup. (**a**) Recordings of single cages (i.e., SCg). (**b**) Recordings of joined cages(i.e., JCg).

To guarantee spontaneous (i.e., without elicitation from the other sex) *H. halys* calling activity, we kept the males and females separated and recorded them in distinct cages (SCg). On the other hand, to assess whether the presence of nearby opposite sex stimulated calling, we made another setup where a male’s cage was joined to a female’s cage (JCg). Joining the opposite sex cages was achieved by linking two sides of male and female cages (Fig. 6) using a clip, which guaranteed that vibrational signals were perceived by the insects without leading them to copulation. In this trial, odors (e.g., pheromones or other volatiles) could freely travel in the air through the cages and physical contact (on the net) was not prevented, thus simulating a natural condition. Given the impossibility of copulation within sexed cages (SCg and JCg), the vibrational communication could be artificially prolonged compared to natural conditions. Therefore, *H. halys* adults were also recorded within mixed-sex cages as control (CCg) to ascertain whether our single-sex cages contained vibrational peculiarities associated with impossibility to achieve mating. The cumulative calling (i.e., Signal 1 and Signal 2) and walking periods were calculated per recording. This was obtained by calculating the sum of the total period of each activity indicator of interest (i.e., Signal 1, Signal 2, and Walking) within each recording. In the case of recording separate cages, a laser Doppler vibrometer (Polytec PDV 100, sensitivity = 5 mm/s/V) was used. When recording the joined cages, two laser Doppler vibrometers (Ometron VQ-500-D-V and Polytec PDV 100) adjusted to the same sensitivity were used simultaneously. Each was pointed at the reflective tape stuck to the upper net of the respective cage (s) and connected to a LAN-XI data acquisition system (type 3050-B-040, Brüel & Kjær sound and vibration A/S) (Fig. 6). When using two lasers, both outputs were assessed simultaneously for differences in amplitude to avoid accounting for a certain signal twice, and to distinguish the source of the signals (i.e., the male cage or the female cage). The cages were placed on an anti-vibrational table (Astel s.a.s, Ivrea, Italy) during recordings. When the cages were not being recorded, they were placed at least 50 cm away from one another in their respective climatic chambers (i.e., each cage on a different rack), to exclude substrate-borne vibrational interference prior to the recordings. All recordings took place between 0800 and 1700 hours. In the 25 °C treatment, the insects were either recorded in their respective rearing climatic chamber or in the biotremology laboratory at Fondazione Edmund Mach (San Michele all’Adige, Italy) inside an acoustic insulated chamber at 25 °C. The insects in the other two temperature treatments were recorded in their respective climatic chambers. The recordings were digitized and stored at a 48kHz sample rate and 24-bit resolution on a laptop computer (HP, EliteBook 8560 p) using “B&K connect” software (Brüel & Kjær sound and vibration A/S). When necessary, the collected data were filtered for noise reduction using the Audacity software (Softonic International, Barcelona, Spain). The recordings were then analyzed using Raven Pro 1.6.1 (The Cornell Lab of Ornithology, Ithaca, NY, USA). Signal 1 cues were selected manually whereas Signal 2 and walking noise were identified through the batch detector feature by specifying the values of the relevant parameters (e.g., minimum or maximum frequencies) (Table S2). Further, to ensure the accuracy of the batch detector, all false detections or undetected signals and walking noise were removed or added manually. The duration of each recording was fixed at 10 minutes. Data acquisition lasted for four months, from February to May 2021. The cages were repeatedly recorded at intervals of two to three days until the insects died.

### Statistical analyses

Statistical analyses were all performed using R version 4.1.1 (R Core Team 2021, Vienna, Austria). The effect of copulation inhibition (JCg) on the vibrational calling and motility of *H. halys* compared to the other cage state categories, was analyzed using Wilks’ Lambda type nonparametric multivariate inference with the package “npmv”^44^. The same package was used to identify significant subsets of variables and factor levels that controlled the family wise error rate, which served as a post hoc analysis. Signals 1 and 2 were considered in the analyses without sex attribution (i.e., MS1, MS2, FS1, and FS2). To assess the differences between male and female signaling within the different cage states, a Kruskal–Wallis test was conducted. The influence of temperature on the cumulative calling periods of each type of signaling activity (i.e., signal 1 and signal 2) and motility (vibrational noise associated with walking) was assessed by fitting zero-inflated generalized linear mixed models (GLMM) with a linear parameterization negative binomial error dispersion for signal 1 and a Tweedie dispersion for signal 2 and walking. To overcome pseudo-replication, the factor “cage number” was used as a random factor in all models which were calculated using the “glmmTMB” package^45^. The covariates were optimized using a stepwise algorithm. For signals 1 and 2, the explanatory variables were temperature, sex, and cage state, whereas for motility, the explanatory variables were temperature and cage state (Table 2). The residual distribution and fitness of the models were evaluated using the DHARMa package^46^. The Akaike information criterion (AIC)^47^ was used to select the best-fitting model. Data exploration plots were built using “tidyverse” package^48^.

Overall survival (OS) was described using Kaplan-Meier curves^49^ for the three temperature categories (10 °C, 18 °C, and 25 °C) for each sex. To simulate the time until insects’ death, Cox’s proportional hazards (PH) model (Cox, 1972) was performed only for males using the median survival time and temperature categories, with “10 °C” as the reference category (negative control). The Cox PH model was then adjusted considering the repetition of insects by cages, as well as a 2-sided 5% significance level. For females, the proportional hazard assumption was not met in the model, indicating that a single hazard ratio would not be adequate to represent the effect of temperature on their survival. Survival analyses were done following Moore^50^ with packages ‘survival’^51^ and ‘survminer’^52^. AIC^47^ was used to select the best-fitting model.

### Supplementary Information

Supplementary Information.

## Data Availability

All the relevant data are presented in the manuscript. The datasets generated and analyzed during the current study are available from the corresponding author upon request.
