# Author Correction: The post-diapause vibrational behavior, motility, and survival of the brown marmorated stink bug *Halyomorpha halys* (Stål) adults at different temperatures

**DOI:** 10.1038/s41598-024-53466-6

**Published:** 2024-02-08

**Authors:** Jalal M. Fouani, Marica Scala, Valentina Zaffaroni‑Caorsi, Vincenzo Verrastro, Gianfranco Anfora, Valerio Mazzoni

**Affiliations:** 1 Center Agriculture Food Environment, University of Trento, 38010 San Michele All’Adige, Italy; 2 Department of Environmental and Earth Sciences, University of Milano Bicocca, 20126 Milan, Italy; 3 CIHEAM Bari - International Centre for Advanced Mediterranean Agronomic Studies, Via Ceglie 9, 70010 Valenzano, Italy; 4 Research and Innovation Centre, Fondazione Edmund Mach, 38010 San Michele All’Adige, Italy

Correction to: *Scientific Reports* 10.1038/s41598-023-50480-y, published online 12 January 2024

The original version of this Article contained an error in Affiliation 3, which was incorrectly given as ‘Department of Mediterranean Organic Agriculture, Mediterranean Agronomic Institute of Bari (CIHEAM Bari), 70010, Valenzano, Italy’. The correct affiliation is listed below.

CIHEAM Bari - International Centre for Advanced Mediterranean Agronomic Studies, Via Ceglie 9, 70010 Valenzano, Italy.

Additionally, Affiliation 4 contained an error and was incorrectly given as ‘Research and Innovation Centre and Technology Transfer Centre, Fondazione Edmund Mach, 38010, San Michele All’Adige, Italy’. The correct affiliation is listed below.

Research and Innovation Centre, Fondazione Edmund Mach, 38010, San Michele All’Adige, Italy.

The original Article has been corrected.

