# Supplementary material for: The post-diapause vibrational behavior, motility, and survival of the brown marmorated stink bug Halyomorpha halys (Stål) adults at different temperatures
Source: Sci Rep. 2024 Jan 12;14:1198. doi: 10.1038/s41598-023-50480-y (PMC10786867; doi:10.1038/s41598-023-50480-y)
Supplement: Supplementary file 1 — Supplementary Information. [file 41598_2023_50480_MOESM1_ESM.pdf]

The post-diapause vibrational behavior, motility, and survival of the brown marmorated stink bug *Halyomorpha halys* (Stål) adults at different temperatures

Jalal M. Fouani, Marica Scala, Valentina Zaffaroni-Caorsi, Vincenzo Verrastro, Gianfranco Anfora, & Valerio Mazzoni

Supplementary materials

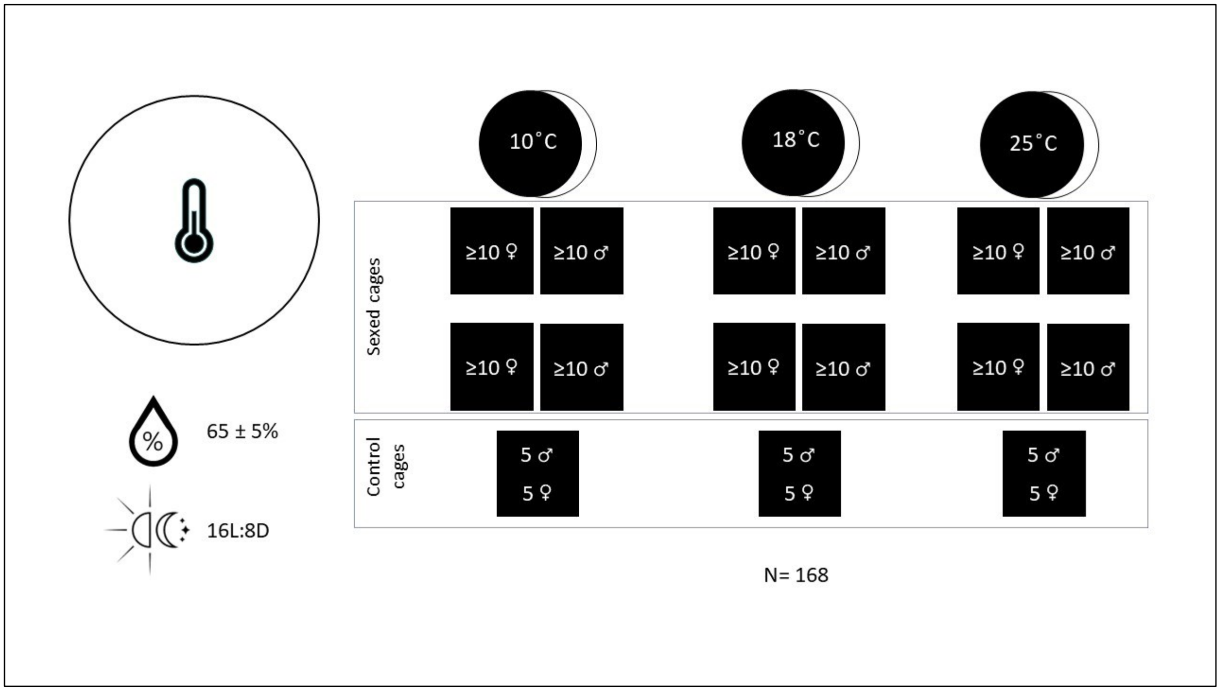

Figure S1. The total sample size used for the experiment and the allocation of sexed insects for each temperature treatment. The sexed cages contained at least 10 insects (maximum of 12 insects), whereas control cages (CCg) contained 5 males and 5 females each. The recording of single (SCg) and joined (JCg) cages was performed on the sexed cages.

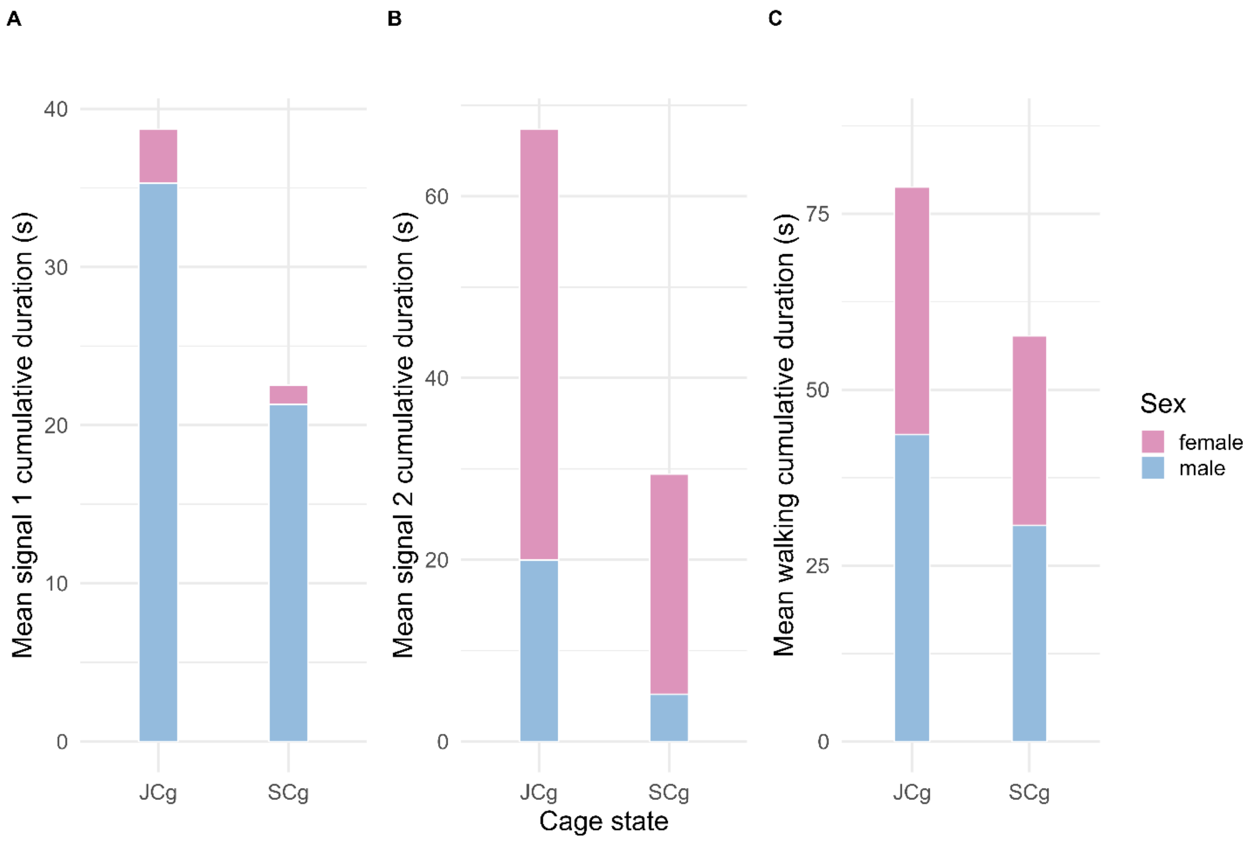

Figure S2. The differences in emitting (A) signal 1, (B) signal 2, and in (C) walking for males and females in joined (JCg) and separated cages (SCg). Females spent significantly higher cumulative periods emitting signal 1 in JCg compared to SCg ($\chi2 = 12.21$, df=1, $p< 0.001$). Both females ($\chi2 = 10.46$, df=1, $p< 0.001$) and males ($\chi2 = 7.1891$, df=1, $p< 0.01$) spent significantly higher cumulative periods emitting signal 2 in JCg. Only males spent significantly higher walking cumulative periods in JCg ($\chi2 = 4.23$, df=1, p=0.04).

Table S1. Survival table of Halyomorpha halys among three different categories of temperature for each sex.

†: Days. NA: Not Available. OS: Overall Survival.

Sex	Temperature	Number at risk (Number of events)	Median OS† (95% CI)
Males	10°C	17 (17)	23 (23 - NA)
Males	15°C	15 (15)	18 (18 - 21)
Males	24°C	18 (18)	4.5 (3 - 8)
Females	10°C	21 (21)	23 (23 - NA)
Females	15°C	16 (16)	18 (14 - 26)
Females	24°C	20 (20)	3 (3 - 5)

Table S2. Parameters' input for the batch detector feature of Raven Pro 1.6.1. (A) represents the input for Walking detection whereas (B) represents the input for Female signal 2 (FS2) detection.

**(A)**			**Walking parameters**		
"Target" window	Min freq	300 (Hz)	"Noise" window	Min occupancy	80%
	Max freq	4096 (Hz)		SnR threshold	7.0 above
	Min duration	0.03125 (s)		Block size	3 (s)
	Max duration	3 (s)		Hop size	2 (s)
	Minimum Separation	0.03125 (s)		Percentile	20.0

**(B)**			**FS2 parameters**		
"Target" window	Min freq	20 (Hz)	"Noise" window	Min occupancy	70%
	Max freq	240 (Hz)		SnR threshold	6.0 above
	Min duration	0.09375 (s)		Block size	3 (s)
	Max duration	1.8125 (s)		Hop size	2 (s)
	Minimum separation	0.125 (s)		Percentile	20.0
